# Therapies for bronchiectasis: mechanisms and potential effects

**DOI:** 10.3389/fimmu.2026.1735598

**Published:** 2026-02-18

**Authors:** Long Yu, Hong-rui Zhu, Xue-hang Jin, Jin Ding, Ting Jin

**Affiliations:** 1 Department of Respiratory and Critical Care Medicine, Affiliated Jinhua Hospital, Zhejiang University School of Medicine, Jinhua, China; 2 College of Medicine, Jinhua University of Vocational Technology, Jinhua, Zhejiang, China; 3 Department of Gastroenterology, Affiliated Jinhua Hospital, Zhejiang University School of Medicine, Jinhua, Zhejiang, China

**Keywords:** anti-infectious drugs, anti-inflammatory drugs, bronchiectasis, bronchodilators, therapy

## Abstract

Bronchiectasis is a complex and heterogeneous disease, and our current understanding of its pathophysiological mechanisms remains in its infancy. Currently, few specifically approved treatments for non-cystic fibrosis bronchiectasis are applied globally, and most guideline-recommended therapies have limited evidence of efficacy. Consequently, the development of new treatment methods is particularly urgent. To gain insight into new drugs under clinical development for adults with bronchiectasis, we conducted a review of the scientific literature and clinical trial registries. In this review, we discuss in detail the mechanisms and potential effects of these emerging therapies, which focus on their anti-inflammatory effects, enhancement of mucociliary clearance, and anti-infectious effects. Clinical research has revealed significant variability among patients, suggesting the need for a multidisciplinary and comprehensive approach. Future efforts should focus on patient stratification using biomarkers and clinical assessments, with an emphasis on precision medicine. Furthermore, it is essential to develop new antibiotics, innovative neutrophil-targeted therapies, and effective mucus-clearing agents to alleviate patient disease burden and minimize the risk of exacerbations.

## 1 Introduction

**Figure 1:**
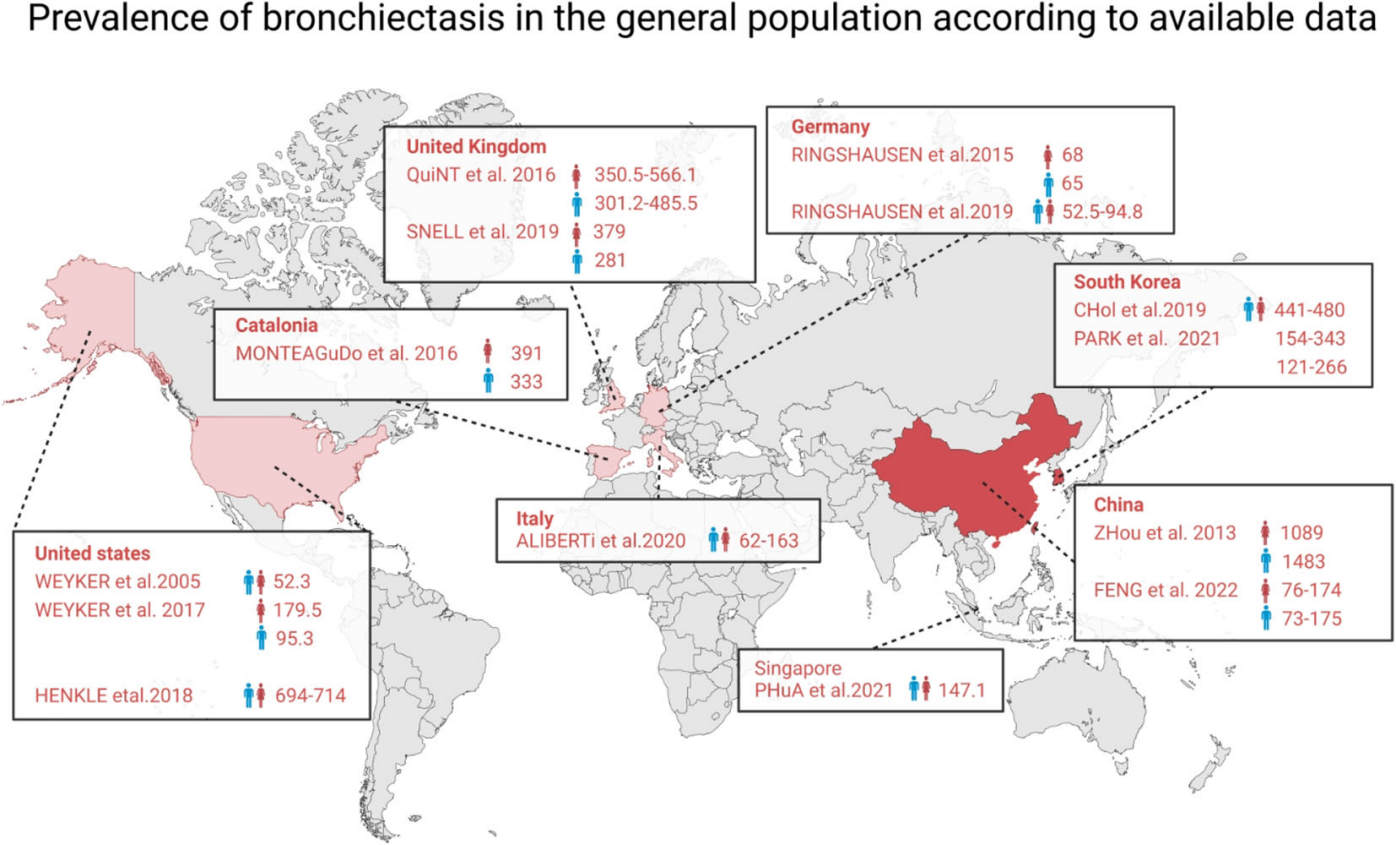
The frequency of bronchiectasis in the overall population based on existing data. Studies from these nations are listed in the corresponding boxes. The data represent the number of individuals impacted by the disease per 100,000 people. The blue and pink symbols denote males and females, respectively.

In the last two decades, the occurrence and frequency of bronchiectasis have notably increased. In the UK, it affects more than 500 people per 100,000 people (1). Comparable or increased rates have been documented in the United States (2), Singapore (3), Germany (4), China (5), and Spain (6) (**Figure 1**). This could be attributed to improvements in diagnostic methods and criteria. Typically, we assess such diseases on the basis of their underlying pathobiological mechanisms and/or response to treatment. Over the past decade, international registries and clinical research collaborations focused on bronchiectasis in adults, adolescents, and children have been established (7–9). These initiatives enhance awareness of the disease and promote research efforts, including the investigation of new treatments and translational science. Consequently, they have led to significant progress in elevating the profile of bronchiectasis. The clinical efficacy of long-term antibiotic and macrolide therapy in reducing disease progression has been well established (10). International guidelines have contributed to the stratification of exacerbations that may benefit from antibiotic treatment and provided management guidance (11). Additionally, novel treatments, including anti-inflammatory drugs that **inhibit** dipeptidyl peptidase-1 (DPP-1), are emerging (12). Cystic fibrosis transmembrane conductance regulator (CFTR) correction therapy has also demonstrated promising results in mucus clearance in CF, but they have not been extensively studied in non-CF bronchiectasis (13, 14). Despite the advancements made over the past decade, numerous ongoing clinical studies have aimed to further elucidate the fundamental principles of bronchiectasis management. In this review, we summarize these clinical studies. Current areas of investigation include the exploration of new drugs, the utilization of large registry databases to characterize endotypes and phenotypes, and the examination of alternative treatments. Future clinical trials may be essential for advancing bronchiectasis treatment by more precisely targeting patient subgroups that are most likely to benefit from specific therapies.

## 2 Pathobiologic mechanisms

**Figure 2:**
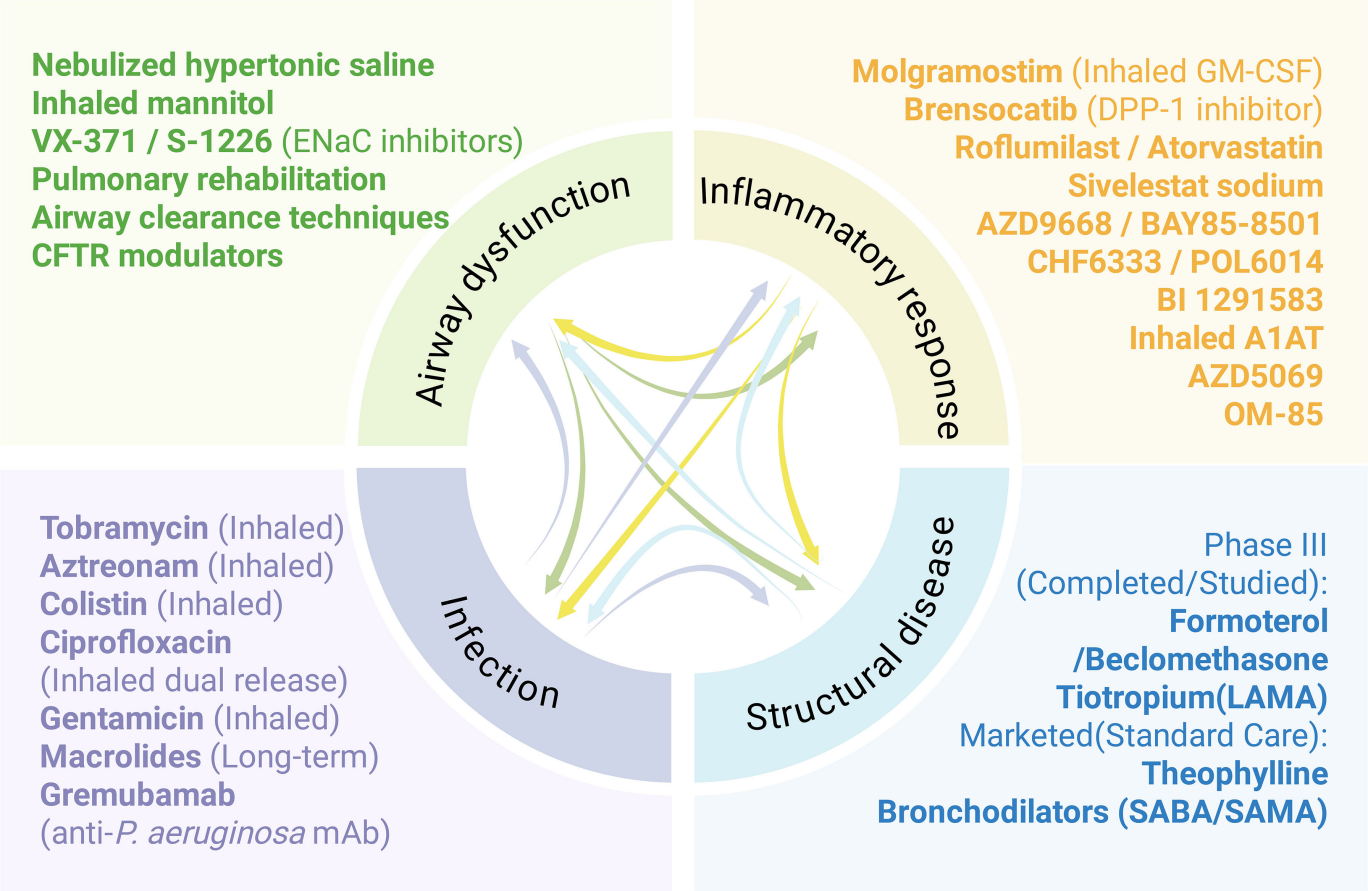
Pathological developmental process of bronchiectasis. The major pathological changes in the bronchiectating airway include mucus hypersecretion and/or blockage, bacterial colonization (including biofilm formation), mucociliary dysfunction, smooth muscle proliferation, and infiltration of inflammatory cells.

Bronchiectasis is a heterogeneous disease. Multiple stimuli play a role in the progression of bronchiectasis, ultimately leading to a vicious cycle of airway remodeling and dilation (15, 16). Initial injury can lead to inflammation, airway disruption and dysfunction, structural disease, and infection. Once this developmental cycle is entered, it progressively worsens and surpasses local and systemic host defense mechanisms. Mucociliary dysfunction leads to impaired mucus clearance, which ultimately results in airway distortion, mucus retention and bacterial colonization. infection causes the recruitment of neutrophils to the bronchial lumen and the infiltration of neutrophils, CD4+ T lymphocytes, and macrophages into the bronchial wall and lung tissue; inflammatory cells activate and release leukotriene B4, TNF-α, IL-8, IL-10, endothelin-1, etc.; **hence bronchiectasis has often been viewed as having Th1 inflammatory patterns.** Inflammatory factors and inflammatory mediators further promote the infiltration of inflammatory cells, causing the “cascade effect” of inflammation. Neutrophil elastase appears to be a key marker. It is now, however, recognized that approximately 20% of patients with bronchiectasis also have significantly elevated sputum eosinophils and Th2 markers (17) (**Figure 2**).

Initial stimulus factors vary from person to person; however, in nearly 40% of people, the cause is unknown (18, 19). Postinfectious bronchiectasis (such as after severe respiratory viral infection or pneumonia) is the most common type. Other causes include autoimmune diseases, chronic obstructive pulmonary disease (COPD), asthma, tuberculosis, fungal infections and genetic disorders (20). Genetic disorders encompass a range of conditions, such as cystic fibrosis, Mounier–Kuhn syndrome, primary ciliary dyskinesia and alpha-1 antitrypsin deficiency. Within the realm of autoimmune diseases, patients may suffer from rheumatoid arthritis, Sjögren’s syndrome, or inflammatory bowel disease; thus, these patients may also present to rheumatologists and gastroenterologists. Furthermore, patients with immune deficiency syndromes, particularly those with common variable immunodeficiency or human immunodeficiency virus infection, may also be at risk for developing bronchiectasis (21). Sequencing the genomes of individuals suffering from idiopathic bronchiectasis might provide a deeper understanding of disease development and reveal possibilities for early intervention.

## 3 Treatment strategy for bronchiectasis

**Figure 3:**
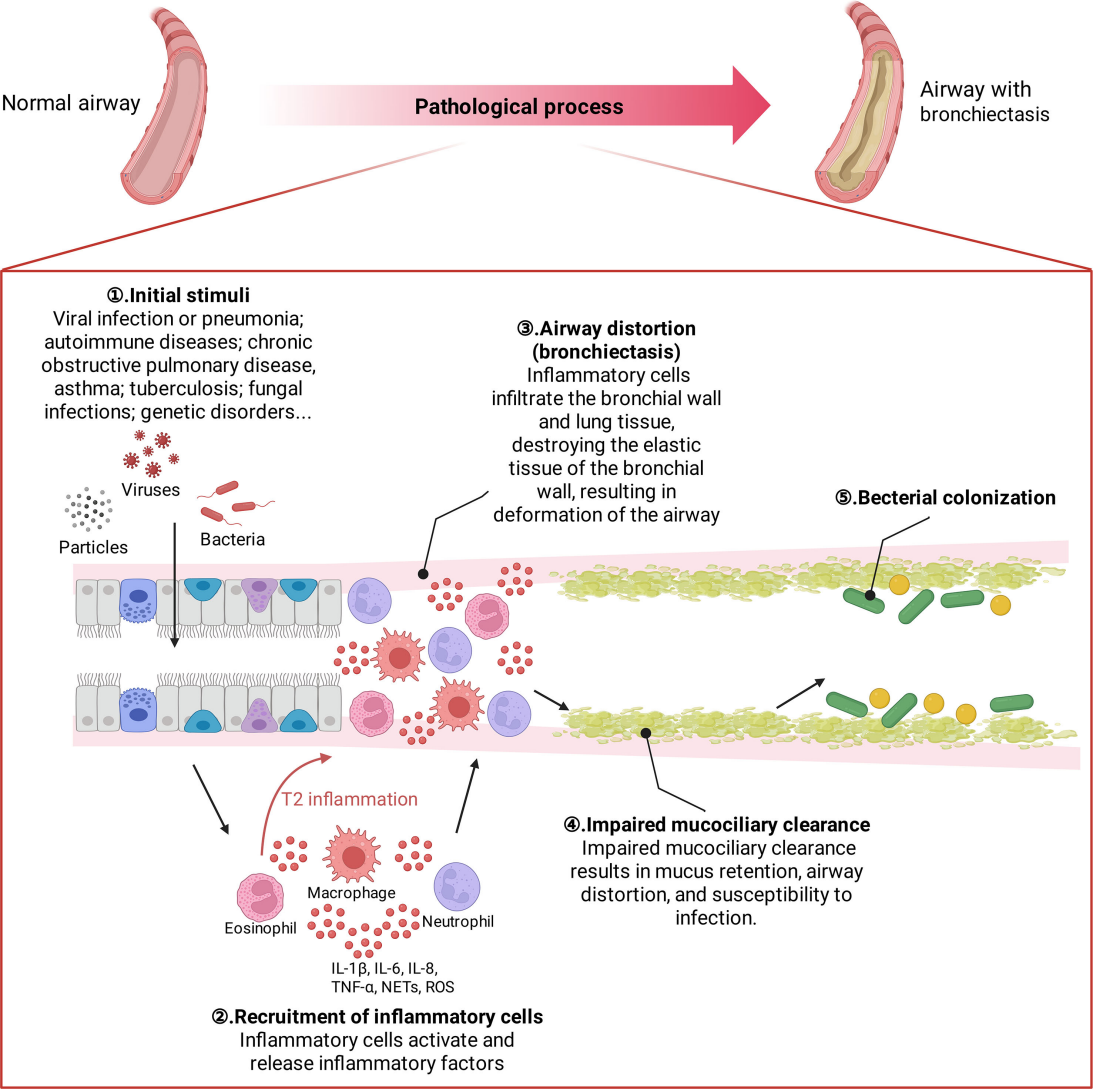
The drug development pipeline for bronchiectasis is categorized by various aspects of the ‘vicious spiral’ model of pathogenesis.

The symptoms associated with bronchiectasis are complex and varied, necessitating a holistic and individualized treatment approach. Given that recurrent respiratory infections, ongoing inflammation, damage to the airways and mucociliary dysfunction represent the four primary elements of the detrimental cycle observed in individuals with bronchiectasis, each pathophysiological step influences the others. This process can be more effectively illustrated via the concept of vortex (16) (**Figure 3**). The vortex model may elucidate why a single treatment, such as antibiotics or anti-inflammatory drugs, yields only modest improvements in the clinical outcomes of bronchiectasis. For example, rather than interrupting the vicious cycle that could be anticipated to halt disease progression, antibiotics focus mainly on a single element of the cycle, suggesting that inflammation and lung injury might result or persist due to additional triggers. Pharmacological interventions targeting numerous pathways could be effective for the treatment of bronchiectasis. Expectorants work on the mucus present on the surface of airways, increasing osmotic pressure, promoting mucus hydration, and facilitating expectoration (22). Antibiotics can reduce the bacterial load in airways or assist in eliminating pathogenic bacteria such as *Pseudomonas aeruginosa*, thereby inhibiting airway inflammation (23). Additionally, anti-inflammatory drugs can mitigate inflammation by targeting neutrophils and potentially eosinophils (**Figure 3**). The following sections introduce the latest advancements in drugs that target various pathological mechanisms.

### 3.1 Anti-inflammatory drugs

The primary cell type involved in the inflammatory process of bronchiectasis is neutrophils, although eosinophils, lymphocytes and macrophages also contribute significantly to the disease (24, 25). Patients with bronchiectasis exhibit a chronic inflammatory reaction in the airways characterized by the accumulation of neutrophils and the subsequent secretion of various inflammatory mediators (26). Upon activation, many neutrophils release a range of proteases, reactive oxygen species, cytokines, and other mediators, leading to significantly elevated levels of inflammatory mediators in the sputum of these patients (27–29). Owing to the significant link between neutrophilic inflammation and bacterial infections, focusing on neutrophils has become a potential treatment strategy for individuals who suffer from recurrent bronchiectasis episodes; this method is presently being explored in clinical studies. Additionally, various methods for suppressing inflammation through immune regulation have yielded positive outcomes. A summary of these treatments is provided below.

#### 3.1.1 Targeting neutrophils

Neutrophil elastase (NE) has the potential to serve as a direct biomarker for neutrophilic inflammation in predicting the risk of future exacerbations (24), although it is not yet widely utilized in daily clinical practice. As a major protease with significant hydrolytic activity, NE plays a crucial role in the progression of bronchiectasis. Studies have confirmed that NE is overexpressed in bronchiectasis, representing a key risk factor for the development of structural lung damage and decreased lung function (30). Additionally, NE is associated with airway mucus hypersecretion and impaired mucociliary clearance (31), both of which are critical risk factors for bacterial colonization and contribute to the foundation of chronic inflammation (32). NE plays a crucial role in the onset and progression of bronchiectasis through various pathways. Even a single NE inhibition strategy can impact the entire complex protease network. As a result, NE is considered a potential therapeutic target, and several NE inhibitors have been developed. Sivelestat sodium is the only NE inhibitor currently approved for clinical use and is utilized primarily in patients with COVID-19 in China (33). However, there is a notable lack of relevant research concerning its application in bronchiectasis. AZD9668, an oral NE inhibitor, has undergone a phase II trial that failed to demonstrate a significant difference in sputum NE activity between the experimental and observation groups post-treatment. Nonetheless, the study indicated that lung function in the experimental group improved somewhat, and sputum inflammatory marker levels tended to decrease (34). BAY85-8501, which is highly selective and specific for NE, did not result in any adverse reactions during the phase I trial. In the phase IIa clinical trial, two groups of bronchiectasis patients were administered either a placebo or BAY85-8501. The findings revealed no significant changes in sputum NE activity or lung function between the two groups following treatment; however, blood NE activity in the treatment group decreased significantly (35). Researchers have hypothesized that the oral administration method may lead to insufficient drug concentrations in the lungs, contributing to low clinical efficacy. Consequently, they developed an inhaled NE inhibitor. CHF6333 is an NE inhibitor delivered as a dry powder inhaler and offers opportunity to assess the importance of higher lung concentrations than serum. *In vitro* experiments have demonstrated that CHF6333 exhibits high selectivity for NE (36). A phase Ib trial of CHF6333 in patients with cystic fibrosis and bronchiectasis concluded in 2021; however, no data are currently available (NCT04010799: 2019-07-08; US Clinical Trials Registry). Additionally, a new clinical trial is currently recruiting participants (NCT06166056). POL6014 is a novel inhaled inhibitor that has undergone phase I trials, which indicated that it is safe, well tolerated, and achieves high drug concentrations in the lungs. Furthermore, it has been shown to inhibit NE activity in sputum from patients with cystic fibrosis following a single administration (37).

Serendex has developed a nebulizer containing Molgramostim, a non-glycosylated form of recombinant human granulocyte-macrophage colony-stimulating factor (GM-CSF). While most therapies aim to downregulate neutrophils to prevent protease damage, inhaled GM-CSF aims to restore or enhance neutrophil bactericidal function, which may be beneficial for clearing persistent infections like NTM A phase 1 randomized, double-blind, placebo-controlled clinical trial (NCT02468908) involving healthy adults has been completed in the UK. This trial evaluated the safety and tolerability of both single ascending doses and multiple ascending doses of Molgramostim in humans. The results indicated that inhaled Molgramostim was well tolerated by the subjects, demonstrated minimal absorption into the bloodstream, and significantly affected white blood cell counts within the normal range. The most commonly reported adverse event was cough. Patients with bronchiectasis are particularly susceptible to non-tuberculous mycobacterial pulmonary disease (NTM-PD). Additionally, an open-label, single arm pilot trial (OPTIMA) was designed to assess the efficacy and safety of inhaled GM-CSF (Molgramostim nebulized solution) in treating NTM-PD in adults with non-CF bronchiectasis (38). Overall, the study found that the study drug was safe and reasonably well tolerated, suggesting that treatment with inhaled Molgramostim may represent a promising therapeutic option.

Although these data have not fully demonstrated the exact efficacy of NE inhibitors in the treatment of bronchiectasis, researchers continue to explore and optimize the therapeutic strategies for NE inhibitors, including the development of inhaled NE inhibitors to increase the concentration of the drug in the lungs and thus improve the clinical efficacy. Currently, the development of multifunctional drugs that not only block the signaling pathways associated with NE but also possess anti-protease and anti-inflammatory properties and promote airway repair is considered a promising avenue for future research.

#### 3.1.2 Cathepsin C inhibition

A different strategy, as opposed to the conventional inhibition of NE, includes the use of medications that focus on the activation of serine proteases within the bone marrow. Cathepsin C (CTSC), which is also referred to as DPP-1, functions as a lysosomal cysteine protease that plays a crucial role in the maturation of neutrophils. This enzyme is responsible for the cleavage of several key proteins, including NE, cathepsin G, and proteinase 3, during the activation process of neutrophils. As neutrophils mature, the activated serine proteases that they produce are subsequently packaged into granules within these cells. Once mature neutrophils enter the systemic circulation, they are able to carry out their functions in the immune response (39). Consequently, the implementation of CTSC inhibitors has the potential to impede the activity of neutrophil serine proteases within airways, thereby possibly influencing inflammatory responses in these regions. As of June 2020, the U.S. FDA has designated brensocatib, a CTSC inhibitor, as a breakthrough therapy for adult non-cystic fibrosis bronchiectasis. Brensocatib, an oral reversible DPP-1 inhibitor, indirectly inhibits NE activity by blocking neutrophil serine protease activation. A phase II clinical trial for bronchiectasis revealed that patients receiving oral treatment with varying doses of brensocatib experienced a significant reduction in sputum NE activity, as well as an extended time to the first exacerbation, suggesting that brensocatib may offer clinical benefits in reducing inflammation and preventing exacerbations (12). The drug has completed the Phase III development for the treatment of bronchiectasis (NCT04594369). The Phase 3 ASPEN trial results, published in the *New England Journal of Medicine* in 2025, demonstrated that brensocatib significantly reduced the annual rate of pulmonary exacerbations and prolonged the time to the first exacerbation compared to placebo (40). BI 1291583 is a novel, highly effective and selective DPP-1 inhibitor that is currently being investigated as a potential disease-modifying therapy in patients with bronchiectasis (41). Results from recent phase II trials showed that treatment with BI 1291583 can alleviate deterioration in adult patients with bronchiectasis (NCT05238675) (42). Following these promising results, several additional trials are now in progress (NCT05865886, NCT05846230). HSK31858 Is an oral, reversible DPP1 inhibitor developed by Haisco, being tested for the treatment of noncystic fibrosis bronchiectasis. HSK31858 demonstrated good safety and tolerability in phase I clinical trials (NCT05663593) and dose dependently suppressed whole blood NE activity. The phase II of the results have not yet been fully made public and it is currently in phase III clinical trials (NCT06660992). In addition, there are several other DPP-1 inhibitors currently in development. Overall, these **DPP-1 inhibitors**, which act by blocking the maturation and activation of neutrophil serine proteases within the bone marrow, are in different stages of clinical trials. Most have yielded promising results, offering potential new systemic options for reducing the protease burden in bronchiectasis.

Distinct from the upstream mechanism of DPP-1 inhibition, **alpha 1-antitrypsin (A1AT, also known as alpha 1-protease inhibitor)** functions as a direct, competitive inhibitor of neutrophil elastase (NE). It is currently being investigated for its potential to directly neutralize active proteases in the airways, mitigate inflammation, and potentially enhance neutrophil function (NCT05582798). This direct protease-inhibition strategy provides a complementary approach to targeting the inflammatory cascade in patients with bronchiectasis.

#### 3.1.3 Inhibiting chemotaxis

A different strategy to alleviate neutrophilic inflammation focuses on directly reducing the influx of neutrophils into affected tissues. The chemokine receptor CXCR2 is pivotal in this process, as its activation by chemokines like CXCL1 and CXCL8 facilitates neutrophil migration. Research has demonstrated that CXCR2 antagonism, as exemplified by the oral CXCR2 antagonist AZD5069 in a clinical trial targeting CXCR1, effectively reduces neutrophil recruitment to the lungs without impairing their immune functions. Specifically, in a study with bronchiectasis patients receiving AZD5069 at 80 mg twice daily for 28 days, a significant 69% reduction in sputum neutrophil counts was observed compared to a placebo control group (43). While exacerbations were reported in both groups without significant difference, four cases of treatment discontinuation due to infection (one pneumonia and three bronchiectasis exacerbations) were noted in the AZD5069 group. Additional studies with CXCR2 antagonists in healthy volunteers and nonhuman primates demonstrated a rise in in blood cytokines, including CXCL1, CXCL8 and CXCR2 ligands, post-treatment, though the clinical significance of this increase is unclear. Furthermore, the 28-day treatment duration may be insufficient to assess all clinically significant effects, highlighting the need for larger-scale studies. Notably, other CXCR2-targeting compounds did not yield positive results in clinical trials (NCT03250689). Alternatively, Roflumilast, a phosphodiesterase 4 (PDE-4) inhibitor, reduces PDE-4 production, suppresses neutrophil chemotaxis and migration, and promotes cell apoptosis for anti-inflammatory effects (44). Currently, its anti-inflammatory effect in stable noncystic bronchial fibrosis is being evaluated in a phase II trial for 12 weeks (NCT04322929) in single-arm, open label, study of 42 patients; no results are available yet.

#### 3.1.4 Targeting eosinophils

Approximately 20% of patients with bronchiectasis, excluding those with asthma, allergic bronchopulmonary aspergillosis, or chronic sinusitis with nasal polyps, exhibit eosinophilia (defined as > 3% or 300/μL), a condition referred to as eosinophilic bronchiectasis (45). Notably, eosinophilic inflammation in bronchiectasis does not imply the absence of neutrophil inflammation (46). The precise role of eosinophils in these patients remains unclear. It is hypothesized that eosinophils may possess bactericidal and antiviral properties against the pathogenic microorganisms commonly associated with bronchiectasis. This study revealed a correlation between blood eosinophil levels and the microbiome. Elevated eosinophil levels are associated with accelerated progression of *Pseudomonas aeruginosa* infection, whereas low eosinophil levels are predictive of severe bronchiectasis and an increased risk of mortality (45), thereby establishing new indicators for disease subtypes. Patients diagnosed with eosinophilic bronchiectasis frequently require inhaled corticosteroids (47); however, the role of Th2-dominant (eosinophilic) inflammation is becoming increasingly recognized (45). Consequently, other anti-Th2 drug treatments have demonstrated greater efficacy (48). In addition, benralizumab is an interleukin-5 receptor-directed cytolytic monoclonal antibody that induces direct, rapid, and massive eosinophil depletion through antibody-dependent cytotoxic activity (49). Retrospective studies have shown a certain relieving effect of benralizumab on bronchiectasis (50).

#### 3.1.5 Vaccine

Commonly used injectable vaccines include the influenza vaccine and the pneumococcal vaccine. Influenza vaccination can prevent secondary lung infections caused by the influenza virus. Although there is a lack of robust specific evidence regarding the benefits of the influenza vaccine for patients with bronchiectasis, indirect evidence suggests that annual influenza vaccination can reduce mortality and morbidity and reduce healthcare costs in high-risk groups (51). A randomized controlled study demonstrated that the administration of the 23-valent pneumococcal vaccine can decrease the frequency of acute exacerbations (52). Furthermore, a study conducted by Japanese researchers involving 105 patients with chronic respiratory diseases, including 20 patients with bronchiectasis, revealed that vaccination with both the influenza vaccine and the 23-valent pneumococcal vaccine can prevent lower respiratory tract infections in these patients (53). Consequently, the bronchiectasis guidelines from the United Kingdom and Saudi Arabia, along with the expert consensus in China, recommend vaccination for patients to mitigate the onset of bronchiectasis (54, 55).

#### 3.1.6 Bacterial lysate

The bacterial lysate OM-85 BV is an immunomodulator widely utilized to prevent acute attacks or exacerbations of COPD, chronic bronchitis, respiratory infections, chronic sinusitis, and other related conditions (56). The primary components of OM-85 BV include freeze-dried lysates from eight of the most prevalent pathogenic bacteria associated with respiratory tract infections, namely, *Haemophilus influenzae*, Diplococcus pneumoniae, *Klebsiella pneumoniae*, Klebsiella odorifera, *Staphylococcus aureus*, Viridans streptococci, *Streptococcus pyogenes*, and Neisseria catarrhalis. Currently, small-sample observational studies have confirmed the preventive effects of Panfusu in patients with bronchiectasis. Furthermore, a double-blind, placebo-controlled randomized clinical trial assessing the efficacy of OM-85 BV in preventing acute attacks in Chinese patients with bronchiectasis is underway (NCT01968421). The results of this study aim to substantiate its preventive effects on acute bronchiectasis attacks through evidence-based medicine (57).

#### 3.1.7 Statins

Recent studies have confirmed the immunomodulatory effects of statins (58). Notably, high-dose atorvastatin has been shown to significantly reduce cough in bronchiectasis patients who do not have a *Pseudomonas aeruginosa* infection (59). Bedi et al. conducted a double-blind, crossover randomized controlled trial demonstrating that atorvastatin can alleviate patient symptoms by reducing systemic inflammation in individuals with bronchiectasis infected by Pseudomonas aeruginosa (60). (NCT01299194). However, this study had several limitations, including low statistical power for secondary efficacy endpoints, a short study duration, and a lack of matching between the two groups. Despite these limitations, the results support the potential benefits of statins as anti-inflammatory agents in patients with chronic bronchiectasis infections. Nonetheless, longer and larger multicenter studies are necessary to further validate these findings.

### 3.2 Anti-infectious drugs

*Pseudomonas aeruginosa* (PA) is one of the primary pathogenic bacteria affecting bronchiectasis patients, and its infection or colonization significantly impacts both the quality of life and prognosis of these patients (61–63). Research indicates that PA infection elevates the risk of all-cause mortality and disease progression and exacerbates psychological distress and cough symptoms (64–66). Several studies have revealed that patients with PA infection experience more extensive damage to their lung lobes and have poorer lung function (67–69). Prospective cohort studies have demonstrated that these patients face a significantly heightened risk of hospitalization (70). PA is adept at forming biofilms and is highly resistant to carbapenem antibiotics. Furthermore, multidrug resistance is prevalent, which complicates treatment efforts.

Since 2000, numerous studies have investigated the clinical application of inhaled antibiotics in patients experiencing frequent exacerbations of bronchiectasis. These studies typically focus on bronchiectasis associated with the long-term use of inhaled antibiotics in the treatment of cystic fibrosis. However, the overall results of these studies have not met expectations. Specifically, while inhaled gentamicin and inhaled colistin have demonstrated potential, clinical trials involving inhaled tobramycin, aztreonam, and ciprofloxacin (both dry powder and liposomal formulations) have failed to achieve the predefined study endpoints (71–77). These unsatisfactory outcomes may stem from limitations in study design, the implementation of cyclic treatment regimens, or the inherent heterogeneity of bronchiectasis itself (78). To date, none of these inhaled antibiotic treatments have received formal approval from regulatory agencies, despite some indications of clinical promise.

Retrospective studies on *Pseudomonas aeruginosa* have reported initial eradication rates of bronchiectasis via various regimens, including oral, inhaled, and intravenous antibiotics, ranging from 52% to 80%. However, these eradication rates decline significantly after one year, underscoring the importance of timely recognition and clinical intervention (79–81). It remains unclear whether this longitudinal decrease in eradication success reflects treatment-related suppression of infection or whether reinfection occurs following successful eradication. Notably, patient groups with higher bacterial loads have exhibited more favorable responses to inhaled antibiotic therapy (82). Consequently, the British Thoracic Society and the European Respiratory Society recommend in their guidelines that long-term inhaled antibiotic treatment should be considered for patients with chronic *Pseudomonas aeruginosa* infections who experience three or more exacerbations per year (83, 84). A small randomized controlled trial involving 35 patients further corroborated the effectiveness of this strategy; two weeks of intravenous ceftazidime or tobramycin was administered, followed by three months of aerosolized tobramycin. One year later, bacterial culture results were consistently negative in 55% of patients (12). However, the efficacy of this approach against *Pseudomonas aeruginosa* and other emerging pathogens requires further validation.

When bronchiectasis deteriorates, targeted systemic antibiotics are typically introduced, and the choice of administration route (oral or intravenous) must consider the severity of the current condition, the resistance profile of the pathogen, and the potential side effects associated with the antibiotic. International guidelines categorize exacerbations that may benefit from antibiotic treatment and provide management recommendations (11); however, the optimal duration of treatment remains inadequately defined, with a 14-day course generally suggested. Nonetheless, there is ongoing debate regarding treatment duration, particularly for adult patients involved in intravenous antibiotic trials, and the advantages and disadvantages of 7-day versus 14-day treatment regimens warrant further investigation (85). The National Institute for Health and Care Research’s SBIVA study (ISRCTN14057228) will assess the feasibility of shorter treatment durations across the UK.

For patients experiencing significant daily symptoms and frequent attacks (three or more per year), it is particularly important to implement additional treatment strategies aimed at improving quality of life and preventing further lung damage. Meta-analyses indicate that the use of macrolide antibiotics can reduce the frequency of attacks, extend the time to the next attack, and increase patients’ overall quality of life (86). Although the precise mechanism of action remains incompletely understood, macrolides may exert their effects by inhibiting quorum sensing in *Pseudomonas aeruginosa (*87). Commonly employed dosing regimens include azithromycin 500 mg three times per week or 250 mg daily, with studies demonstrating that macrolides are generally safe and associated with acceptable side effects over a one-year period (88). Furthermore, the **BOAT trial (Bronchiectasis: Optimizing Azithromycin prevention Treatment to reduce exacerbations; ISRCTN59922389)**, a pragmatic phase IV randomized controlled trial, is currently investigating the optimization of long-term azithromycin treatment to further refine prevention strategies for exacerbations. Nonetheless, owing to the risks of drug resistance and potential gastrointestinal, cardiac, and auditory side effects, macrolides should be administered with caution, particularly in patients with nontuberculous mycobacterial infections or when such infections have not been conclusively ruled out.

In summary, when considering long-term macrolide therapy or inhaled antibiotic therapy, it is essential to comprehensively evaluate individual patient disease characteristics, drug contraindications, adverse reactions, and other relevant factors to optimize therapeutic outcomes.

### 3.3 Airway clearance therapies

Under normal circumstances, airway mucus is secreted by submucosal glands, goblet cells, and airway epithelial cells, with water being the primary component. Although the mucoprotein (MUC) content is low, it plays a crucial role in the viscoelasticity of mucus. During bronchiectasis, persistent inflammation in the airways occurs, leading to the stimulation of goblet cell differentiation and proliferation by inflammatory cells and mediators, which is often accompanied by submucosal gland hypertrophy. The expression of MUC5AC and MUC5B is upregulated, with MUC5AC influencing the thickness of the mucus gel layer and viscosity affecting the consistency of the mucus sol layer. The hypersecretion of airway mucus is common throughout disease progression and significantly impacts patient prognosis (18, 89–92). In patients with bronchiectasis, airway mucus becomes highly concentrated, suggesting that intervention in mucus hypersecretion may represent a viable target for drug treatment. Airway clearance therapies encompass nonpharmacological strategies, mucus-activating treatments, and pulmonary rehabilitation combined with exercise. The primary objective of these interventions is to mobilize secretions, thereby reducing coughing and dyspnea while preventing further airway damage. Some of the relevant treatments are summarized below.

#### 3.3.1 Drug treatment strategy

Published guidelines identify airway clearance as a critical treatment for bronchiectasis, although the supporting evidence remains relatively weak (83, 84, 93). Research on epithelial dysfunction in bronchiectasis is limited compared with that in cystic fibrosis, where CFTR corrector therapy has shown promising results (94). CFTR mutations are controversial noncystic fibrosis bronchiectasis, with studies showing varying frequencies of these mutations (95–97). However, CFTR dysfunction may play a role in some cases, making CFTR correction a potential treatment option (98). Inhibitors of the epithelial sodium channel (ENaC) represent a potential therapeutic strategy to facilitate mucus rehydration by hindering excessive sodium absorption. While the rationale for ENaC inhibition has been extensively explored in cystic fibrosis (CF) due to its role in CFTR-dependent secretion, it is critical to distinguish these mechanisms from the pathophysiological environment of non-CF bronchiectasis. Clinical evidence in the bronchiectasis population remains limited; for instance, a phase II clinical trial of the ENaC inhibitor VX-371 failed to demonstrate significant improvements in the percentage of predicted forced expiratory volume (ppFEV1), although the treatment was generally well-tolerated. Similar challenges in demonstrating clear clinical benefits have been observed with other agents like BI 1265162, contributing to ongoing debates regarding ENaC as a viable therapeutic target in these populations. A major breakthrough in the management of airway clearance was provided by the CLEAR trial (2025) (99). This landmark study evaluated the efficacy of hypertonic saline versus carbocisteine specifically in patients with bronchiectasis; however, the results demonstrated no significant benefit in exacerbation reduction or quality of life with either the systemic mucolytic or nebulized hypertonic saline. By providing high-quality comparative data for non-CF cohorts, the CLEAR trial underscores the necessity of moving toward evidence-based, precision airway clearance therapies tailored specifically for the bronchiectasis population.

In addition to CFTR and ENaC-related treatments, several novel therapies are currently under investigation. ARINA-1 is a new nebulized product composed of ascorbic acid, glutathione, and bicarbonate that operates through a mechanism independent of CFTR, offering a potential new treatment for mucus clearance disorders in cystic fibrosis (100). Recently, a phase IIa clinical trial of ARINA-1 demonstrated positive results in adult subjects with noncystic fibrosis bronchiectasis (NCFBE) characterized by excess mucus and cough (NCT05495243). Hypertonic saline delivered via nebulization serves as a mucoactive therapy and has shown promising advantages for individuals diagnosed with bronchiectasis. In a limited-scale investigation, researchers reported that daily nebulization of a 7% saline solution led to improvements in lung function and quality of life (101). Furthermore, inhaled dry powder mannitol is utilized in the treatment of cystic fibrosis and has been shown to enhance ciliary clearance, although its precise mechanism of action remains unclear (102, 103). Another innovative biophysical therapeutic agent, S-1226, employs carbon dioxide (CO_2_)-rich air in combination with aerosolized perfluorocarbons (PFOB) to promote airway dilation, enhance mucus clearance, and improve blood oxygenation. Preliminary efficacy results indicate that S-1226 may positively impact the management of cystic fibrosis-related lung disease; however, further clinical trials are necessary to validate these findings (NCT03903913). Finally, CSL-787 is a nebulized immunoglobulin formulation currently in development for the treatment of noncystic fibrosis bronchiectasis (NCT04643587). The emergence of these new therapies offers renewed hope for the management of bronchiectasis and cystic fibrosis.

#### 3.3.2 Nonpharmacological methods

Nonpharmacological methods of airway clearance include active recirculation breathing techniques, autogenic drainage (which involves a controlled expiration rate and depth to facilitate secretion mobilization), slow expiration with the glottis open in the lateral decubitus position, and the application of positive expiratory pressure. Additionally, oscillation devices and high-frequency chest wall oscillation are utilized (21). The advantages of these treatments lie in their independence for patients, as they are generally safe and straightforward to learn (104). Observational studies focusing on pulmonary rehabilitation and exercise programs carried out in both Australia and Italy have demonstrated significant improvements in various physiological measurements, including the 6-minute walk distance and quality of life related to health (105, 106).

## 4 Bronchodilators

Some patients with bronchiectasis present clinically with airflow limitation or dyspnea due to structural damage (107). Current guidelines from the European Respiratory Society and the British Thoracic Society recommend trials of bronchodilator therapy (83, 84), although robust evidence supporting their efficacy is lacking. The effectiveness of theophylline as a bronchodilator remains unproven. A completed Phase 4 clinical trial evaluated the safety and clinical effectiveness of theophylline for treating bronchiectasis that is not related to cystic fibrosis; however, the findings have not yet been released (NCT01684683). Consequently, there is currently no evidence to support or refute the use of theophylline in treating bronchiectasis. Studies have indicated that other bronchodilator treatments can enhance lung function (108); for example, the combination therapy of formoterol and budesonide can significantly alleviate symptoms and enhance the quality of life for patients (109). A two-way crossover trial indicated that tiotropium improved forced expiratory volume in 1 second, although it did not lead to a reduction in exacerbation frequency (110). Additionally, a Phase III clinical trial evaluating the combination of formoterol and beclomethasone in the treatment of bronchiectasis is currently recruiting participants (NCT03846570). While the sample sizes of existing trials are relatively small, they provide preliminary evidence of the benefits of bronchodilators in improving lung function and alleviating dyspnea. However, it remains unclear how effective they are in diminishing the occurrence of exacerbations and improving quality of life. Future studies are needed to better identify minimal clinically important differences in key lung function variables and phenotypic characteristics that predict treatment response. The difficulty of establishing the efficacy of bronchodilators in this heterogeneous population is further exemplified by the premature termination of a recent major clinical trial (111). This termination underscores the ongoing challenges in recruitment and the potential lack of clinical benefit for unselected patients, reinforcing the necessity for precision medicine to identify specific phenotypes that may respond to bronchodilator therapy.

## 5 Conclusion

While some progress has been made in the research and treatment strategies for bronchiectasis, no officially licensed specific treatment has yet been developed. To address this gap, it is imperative to pursue breakthroughs through an increased number of randomized controlled trials. Specifically, further investigations are needed to identify which patient groups benefit from physical chest clearance, mucus-modulating treatments, and regimens such as inhaled corticosteroids, long-acting beta-agonists, anticholinergics, and anti-inflammatory and antiinfective treatments. This will allow for more precise targeting of patient groups and optimization of the effectiveness of clinical trials. Additionally, further exploration and research are necessary to determine the optimal route of administration and duration of these treatments.

### 5.1 Search strategy

Literature was systematically searched in databases including PubMed and international clinical trial registries (Controlled-Trials.com and ClinicalTrials.gov) from their inception through December 2025. This review focused on articles related to bronchiectasis regarding incidence, therapy, drugs, healthcare, etiologies, and pathobiological mechanisms, excluding those without radiological confirmation. Only adult studies were considered, except for occasional pediatric data in high-risk population studies.
